# Multiple Myeloma as a Bone Disease? The Tissue Disruption-Induced Cell Stochasticity (TiDiS) Theory

**DOI:** 10.3390/cancers12082158

**Published:** 2020-08-04

**Authors:** Jean-Pascal Capp, Régis Bataille

**Affiliations:** 1 Toulouse Biotechnology Institute, University of Toulouse, INSA, CNRS, INRAE, 31077 Toulouse, France; 2 Faculty of Medicine, University of Angers, 49045 Angers, France; fregisbataille@gmail.com

**Keywords:** multiple myeloma, MGUS, oncogenesis, plasma cells, endosteal niche, bone lesion, bone marrow microenvironment, cell-to-cell heterogeneity, gene expression noise

## Abstract

The standard model of multiple myeloma (MM) relies on genetic instability in the normal counterparts of MM cells. MM-induced lytic bone lesions are considered as end organ damages. However, bone is a tissue of significance in MM and bone changes could be at the origin/facilitate the emergence of MM. We propose the tissue disruption-induced cell stochasticity (TiDiS) theory for MM oncogenesis that integrates disruption of the microenvironment, differentiation, and genetic alterations. It starts with the observation that the bone marrow endosteal niche controls differentiation. As decrease in cellular stochasticity occurs thanks to cellular interactions in differentiating cells, the initiating role of bone disruption would be in the increase of cellular stochasticity. Thus, in the context of polyclonal activation of B cells, memory B cells and plasmablasts would compete for localizing in endosteal niches with the risk that some cells cannot fully differentiate if they cannot reside in the niche because of a disrupted microenvironment. Therefore, they would remain in an unstable state with residual proliferation, with the risk that subclones may transform into malignant cells. Finally, diagnostic and therapeutic perspectives are provided.

## 1. Introduction

During the last 25 years, a lot of research has been devoted to multiple myeloma (MM), its basic biology, clinical presentation, and management. These new data have improved the diagnosis, prognosis, and treatment of the disease. The natural history of MM has been clarified: MM occurs from obligatory precursor stages, monoclonal gammopathy of undetermined significance (MGUS), and smoldering MM (SMM), through the malignant transformation of the normal counterparts of MM cells, now identified as long-lived plasma cells which derive from memory B cells and plasmablasts [1]. These cells reside inside the endosteal niche within the bone/bone marrow micro-environment (BME) where they fully differentiate [2,3,4]. Furthermore, extensive studies have characterized MM cells and their capacity to proliferate and differentiate in the close vicinity of the BME through a better knowledge of their genotype, morpho-phenotype, and kinetics [5]. Finally, the mechanisms of lytic bone lesions (LBLs) have been dissected [6]. These changes, considered as the extended phenotype of MM cells, are the hallmark of MM. These specific MM changes also include the partial replacement of normal plasma cells by MM cells inside the bone remodeling compartment. This replacement is responsible for hypo-gammaglobulinemia, another specific hallmark of the disease.

At the present time, major advances on these topics have led to a “standard” model of MM oncogenesis, which is largely agreed on by the scientific community [7,8,9]. According to this model, MM oncogenesis is (i) viewed as a multistep process from normal counterparts to overt disease through MGUS/SMM and (ii) mainly explained by a genetic instability responsible for chromosomal and gene alterations accumulating in normal counterparts anywhere along the lymphopoietic and plasmacytopoietic process. This instability generates MGUS cells followed by MM cells with full malignancy associated with a stronger capacity to survive, to proliferate, and to destroy bone trabeculae, and to invade extra-osseous tissues. However, whereas LBLs are a hallmark of MM, the role devoted by the standard model to these MM-induced bone changes remains a passive one, with LBLs being considered as end organ damages. In this context, it is not satisfying to have a model of MM oncogenesis missing the most specific component of the disease.

Indeed, many recent elegant works and reviews present changes in bone remodeling as an omnipresent component in MM and its precursor steps. More precisely, these works suggest a special relationship between MM cells (and their precursors) and bone cells and matrix along the natural history of MM. This is supported by the following major facts: (i) Within the BME, the osteoblastic/endosteal niche is the normal residence of the normal counterparts of MM cells, a permissive environment to differentiate [10,11,12,13,14,15,16]; (ii) bone fragility turns out to be a morbidity significantly associated with MGUS and maybe with earlier phases (pre-MGUS) [17]; (iii) prolonged osteoblast suppression is now presented as the major mechanism of MM-induced bone changes close to the mechanisms of MM oncogenesis [18].

Overall, these works emphasize bone tissue as a “tissue of significance” in MM [17] that is frequently proposed to a permissive tissue. However, this new context offers opportunities to consider bone tissue as a causal one and thus better modeling MM oncogenesis by reconciling bone changes (as a causal microenvironment) with genetics, which contribute to the major part of the standard model. Is a causal explanation integrating MM genetics and changes in bone remodeling possible? The purpose of the current critical review is to present the tissue disruption-induced cell stochasticity (TiDiS) theory, which is able to integrate these components altogether into a unique and causal explanation of MM oncogenesis. The TiDiS theory can be viewed as an alternative explanation to the overly autonomous “chromosome-centric” standard model. This causal concept strongly supports MM (in particular, but cancer in general) not only as an evolutionary multistep process but also as an ecological (environmental tissue dependent) process in which a critical initiating/promoting role is devoted to an environmental niche of significance and its disruption [19,20,21].

## 2. Standard, Unifying, and Extended Models of MM Oncogenesis

### 2.1. Characteristics of MM and MM Cells

MM is characterized by its natural history from normal counterparts to overt disease through precursor stages, pre-MGUS, MGUS, and SMM. In this history, bone is a tissue of significance. Actually, the natural history of MM from its precursor stages is characterized by an early disruption of the endosteal niche marked by a shift from osteoblastic to osteoclastic presentation [22,23,24,25].

MM cells are characterized by their genotype [26], morphotype (nuclear-cytoplasmic asynchrony, chromatin overture) [27], phenotype (survival, stemness, atavic) [5,28], and kinetics (persisting slow-cycling cells, residual proliferation, lack of full differentiation) [5,29]. These characteristics of MM cells are those of cells “in disruption” with their tissue of reference, the endosteal osteoblastic niche.

LBLs are a hallmark of MM and constitute the extended phenotype of MM cells that is their capacity to destroy bone trabeculae. Actually, two types of bone lesions can be distinguished. First, LBLs occur through specific mechanisms which have been almost fully identified, using ancillary pathways such as Wnt, inhibins, IGF1, as the atavic/survival phenotype [6]. Second, generalized bone loss, pre-existing to MM, is probably due to disruption of the mesenchymal stromal to osteoblastic transition and responsible of the early endosteal niche disruption [17,18,30,31].

### 2.2. Standard Model

At the present time, the scientific community has found an overall agreement on a “standard” model of MM oncogenesis [9,32,33]. It is mainly based on chromosomal alterations of the genotype of MM cells. Indeed, chromosomal instability represents the major genetic alteration encountered in MM as it is the case in the majority of cancers. According to the major genetic and genomic studies of MM cells, three events altering chromosomal ploidy and stability are involved at the origin of MM: (i) Hyper-diploidy (HD), mainly related to trisomies, (ii) non-hyper-diploidy (NHD), related to 14q32 IGH chromosomal translocations, mainly t(4;14), and (iii) a particular driving event, t(11;14), mainly associated to diploidy (D) and involving *CCND1*. Of note, these early chromosomal alterations are not only present in MM cells, but also in the memory B cells of patients with MM, and in MGUS cells [32]. Thus, they seem necessary but not sufficient to generate MM from MGUS.

Subsequent genetic alterations (new chromosomal alterations and point mutations) would be necessary for MM progression. They have been described and thoroughly reviewed [34,35,36], and genetic heterogeneity in MM has also been discussed in recent years [8,37]. Of note, chromosomal alterations reflect different levels of chromosomal instability. MM with t(11;14) are frequently D MM and present with the lowest incidence of del13q, thus with the lowest chromosomal instability [38]. On the contrary, NHD MM, especially those with t(4;14), present with the highest instability, whereas HD MM are in the “just right” situation [39]. Furthermore, both chromosomal alterations and instability significantly correlate with the proliferation and differentiation status of MM cells, their relation to the BME, their capacity to destroy bone trabeculae, and with the natural history of MM. D MM present with the longest history, NHD MM with the shortest, and HD with an intermediary position [5,39,40,41].

As outlined in Table 1, these correlations allow delineating “multiple” types of MM with different evolving pathways. However, despite these correlations which strongly suggest a special relation of MM genetics to the BME, the “standard” model of MM oncogenesis remains an autonomous “chromosome-centric” one. Experts were aware of the lack of a universal/unique driving genetic event at the origin of MM, with the exception of MM with t(4;14) involving the oncogene *MMSET*. For these reasons, they were in search of a (re)unifying event to reconcile MM genetics with the other major characteristics of MM cells.

### 2.3. (Re)unifying Model

The overexpression of both the CCNDs (at least one CCND1, 2, or 3) and Myc proteins in MM cells was considered as this unifying event by all the experts, offering a relevant explanation of the aberrant kinetics of MM cells [39]. Indeed, such overexpression hampers the capacity of targeted B and plasma cells to exit the cell cycle, and is responsible for the residual proliferation of MM cells, which is one of their major characteristics. Such residual proliferation is also facilitated by the aberrant kinome (CD117 or CD221 aberrant expression)/phosphatasome (lack of CD45 expression) ratio of MM cells, which is responsible for an abnormal response of MM cells to growth factors such as IL6 and IGF1 [5]. Of note, this unique and universal residual proliferation, regardless of its mechanisms, is well reflected by the nuclear-cytoplasmic asynchrony universally and specifically observed in MM cells [27].

Such a population of persisting slow-cycling, genetically altered memory B cells and plasmablasts represents an ideal population to be submitted to selection and subsequent evolution, according to a branching evolution model based on genetic instability then natural selection. The strong correlations found between chromosomal ploidy (D, NHD, and HD) and the proliferation and differentiation status of MM cells (Table 1) suggested genetics to be at the origin of this status [39,42]. But paradoxically, CCNDs and Myc overexpression (as CD117/CD221 aberrant expression) are of epigenetics origin in the majority of patients. Thus, by identifying the overexpression of CCNDs (and Myc) as the unifying event of MM, the enlarged “standard” model integrates not only the genotype of MM cells, but also their morpho-phenotype and kinetics (residual proliferation) (Table 2).

However, the fact that the unifying event turns out to be of epigenetic rather than of genetic origin suggests it could also be of environmental origin. Indeed, because the proliferation and final differentiation of the normal counterparts of MM cells are totally dependent on the BME within the endosteal niche, the abnormal capacity of MM cells for a residual proliferation could find its origin in disruption between MM cells and this BME. Although enlarged to the morpho-phenotype and kinetics of MM cells, the “standard” model of MM oncogenesis does not integrate the extended phenotype of MM cells: Their capacity to destroy bone trabeculae and to facilitate MM cell growth through an active/permissive role of the BME.

### 2.4. Extended Model to the BME

There are several strong arguments to extend the model of MM oncogenesis to the BME. MM cells present with the constant capacity to interact with the BME (their extended phenotype). Overall, both normal and malignant plasmacytopoiesis interact with the BME, inside the endosteal niche. These interactions occur all along the natural history of MM from its precursor stages, especially MGUS. In MM, these interactions, especially the specific prolonged osteoblast suppression, are likely close to those of MM oncogenesis [18]. Many types of cells and of soluble factors have been involved in the uncoupling process occurring inside the endosteal niches invaded by MM cells [6]. Among them, the stromal cells, activated in the close vicinity of MM cells, have a pivotal role, directly or indirectly [43,44].

Stromal cells are able to both stimulate osteoclasts through potent osteoclasts activating factors like RANK ligand and to inhibit osteoblasts through inhibins like activin A and other factors (DKK1, sclerostin, GfII…) [45,46]. Furthermore, this “reactive stroma” has the capacity to stimulate MM cell growth, revealing the existence of an unexpected “vicious cycle” between bone and MM [47]. We were the first to show that the most specific mechanism associated with the occurrence of LBL was a prolonged suppression of osteoblasts, of osteo-formation, and of osteocalcin production [48,49,50]. On the contrary, we have shown that osteoblasts activity was maintained (or increased) in the exceptional cases of osteosclerotic MM (and MM lacking LBL), in many solitary myelomas and in SMM, an early stage of MM [49,51,52]. These situations suggest that the maintenance of bone formation, even temporary, can limit tumor progression.

Of note, recent elegant works have extended this suppression of osteoblasts to their lineage related cells, lining cells, and above all osteocytes, altogether within the osteoblasts–osteocytes-lining cell complex [53,54]. Among them, osteocytes appear to play a pivotal role. This role has been extensively and recently reviewed [54,55]. This critical point suggests that the mesenchymal stem cell itself could be involved and that a disruption of the mesenchymal stromal stem cell to osteoblast transition could occur in MM. This is in agreement with the fact that bone as a whole is now considered as a tissue of significance for MM and its precursor stages in the extended model (Table 2).

During the 70s, the experts questioned the association of osteoporosis with MGUS, mimicking MM, and thus initially described as pseudo-MM: Coincidence or real entity [56]? Actually, MGUS is significantly associated with excessive bone fragility (qualitative and quantitative bone abnormalities) and overt osteoporosis [17]. For this reason, the term of MGSS for “monoclonal gammopathy of skeletal significance” has been proposed by Drake [17]. This MGUS-associated bone fragility is probably not induced by MGUS but rather could pre-exist to MGUS (and thus to MM) as an accentuation of the normal senescence of bone tissue [57]. Indeed, comparative studies of normal, senescent, and MM bone marrow stromal cells have revealed that senescence emerges as an important underlying and pre-existing contributor to the prolonged suppression of osteoblast differentiation in MM [18]. Aging, but also various inflammatory and some malignant conditions, induce suppression of osteogenesis and increased adipogenesis [18]. Thus, in this context of permissive or even causal BME, the question is whether age-related changes or changes of other origin in bone marrow stromal cells could contribute to the development of MM and/or its progression from MGUS.

It is worthwhile to note that we have demonstrated the existence of a disruption/shift of the endosteal niche in MGUS at the histological level [22]. Such a “shifted” niche, from a quiescent osteoblastic to a reactive osteoclastic profile could represent a “permissive” or even causal BME for precursor MM cells. This reactive niche, identical to that observed in mice, attracts precursor MM cells in a more acidic, hypoxic, clastogenic, and immunosuppressive milieu than the normal osteoblastic one [58,59], thus facilitating genetic instability within these cells, and impacting the nature of selection/competition between them. To summarize, a disruption of the endosteal niche, with an excess of osteoclasts activity, is observed not only in MM but also in MGUS.

Whereas in overt MM the disruption is due to reactive stroma-induced uncoupling between bone resorption and bone formation, in MGUS it results from the disruption of the mesenchymal stromal to osteoblast transition in relation to (excessive) bone senescence [57], especially mediated by osteocytes [18]. During the malignant transition from MGUS to overt MM, both mechanisms interact to accentuate the disruption, and facilitate the occurrence of the MM bone disease, not only LBL, but also generalized bone loss, the second component of the MM bone disease (Table 3). We and others have previously emphasized that the natural history of MM includes a pre-MGUS phase, characterized by a hyper-gammaglobulinemia, reactive plasmacytosis (excess of circulating plasmablasts) or even transient MGUS in a context of immune deficiency [60,61]. Genetically altered memory B cells and plasmablasts could expand during such a pre-MGUS stage, because they remain sensitive to both polyclonal B cell activation and specific antigenic activation. Indeed, Ig-genes somatic mutations remain active up to the stage of MGUS, whereas such Ig-genes mutations are fixed in MM [62]. MM memory B cells, also genetically altered as are MM cells and able to re-generate MM when inoculated, can be reactivated in a similar way [32]. In this context, the role of the endosteal osteoblastic niche which is the normal residing site of memory cells and plasmablasts to differentiate, as a selection barrier, and of its putative alterations (due to a pre-existing disruption), could be essential to facilitate the transition from transient to permanent MGUS.

## 3. A Microenvironment-Based Model: The TiDiS Theory

### 3.1. General Scheme of the TiDiS Theory

Here, we consider a new vision of the dependency of MM ontogeny and phylogeny on bone remodeling changes because bone is a tissue of significance in the natural history of MM [17] and bone fragility/senescence could be at the origin/could facilitate the emergence of MM [18]. Now many authors suggest that age-related bone disturbances or inflammatory or malignant diseases could play a role in the development of MM in the early phases (not only in progression), or even in initiation [17,18,44].

We would like to go further by providing a theory giving bone/BME a potential role in the initiation of MM, which may ultimately lead to therapeutic perspectives. Several arguments can now be formulated against a unique initiating role of genetic alterations because alterations of the host environment could also contribute to the emergence of tumors [21]. For instance, oncogenic mutations are not sufficient to start transformation; oncogenesis can initiate from disruption of the BME, and cancer cells harboring multiple genetic alterations can be controlled and reverted by a healthy tissue environment [21,63,64,65].

We propose to integrate disruption of the BME (the bone remodeling compartment is our case), differentiation, and genetic alterations in a coherent scheme that starts with the observation that the endosteal niche controls differentiation [66]. From stem and precursor cells characterized by high cellular stochasticity, differentiation, especially among hematopoietic cells, has been associated with a decrease in stochasticity and cell entropy (for a review, see [67]). This phenomenon is especially observed at the level of gene expression, with a homogenization of the expression profiles between cells [67,68,69,70]. This decrease in cellular stochasticity occurs thanks to the establishment of cellular interactions in different developmental systems (for a review, see [71]). When they are disrupted, stochasticity re-increases, which would correspond to a dedifferentiation, and an increase in cell-to-cell heterogeneity [71,72].

Therefore, we can consider that tissue and niche disruption could produce an increase in stochasticity (phenotypic instability) and concomitantly a loss (or lack) of full differentiation which could lead to tumor transformation. This process could only initiate with a loss of the environmental constraints present in the healthy tissue, whatever genetic alterations are present or not in the cells, because they are known to control the level of cellular stochasticity. This TiDiS theory is supported by much experimental evidence that has been reviewed elsewhere [63,64,65,71], especially in the context of MM [60].

### 3.2. The TiDiS Theory in MM

In the context of polyclonal activation of B cells such as polyclonal expansions of plasmablasts, which are reactive plasmacytosis [73], the pools of memory B cells and plasmablasts increase. These cells compete for localizing in endosteal niches with the risk that some of them cannot fully differentiate if they cannot reside into the niche because of a disrupted BME. Therefore, they would remain in an unstable state with relatively high cellular stochasticity and residual proliferation. Thus, if the bone remodeling compartment is already disrupted due to physiological or environmental alterations, more cells would remain non-fully differentiated for a longer time. Thus, this population would be more prone to contain subclones that will transform into stabilized MGUS cells and then into malignant cells.

Regarding genetic abnormalities, several possible cases can be considered: (1) Genetically abnormal B cells preexist and are amplified along with normal B cells; (2) genetically abnormal B cells are generated during the expansion phase; (3) no genetic abnormality is present in any B cell at the end of the activation.

(1) If genetic alterations pre-exist, the disease would develop more quickly, but differently, depending on the type of genetic disorder. It is expected to be more aggressive in the case of t(4;14), and more generally in cases where an oncogene playing on epigenetics, and therefore differentiation, is affected. Nevertheless, these disorders are not sufficient because they are present in healthy cells and patients.
(2) No alteration preexists but the niche disruption is clastogenic and favors the appearance of genetic alterations. Here again, the disease cannot be considered as initiated by genetic modifications and cannot be understood without the initial environmental alteration.

In these cases, the proliferation disorder could be considered of genetic origin. Nevertheless, pre-existing abnormal B cells do not produce MGUS unless polyclonal activation occurs. Moreover, aneuploid B cells are highly frequent in healthy individuals and do not systematically lead to MGUS following polyclonal activation of B cells, suggesting again that non-genetic factors favor MGUS stabilization, especially the disrupted BME that would lead to a permanent pool of non-fully differentiated cells. Thus, it appears that genetic abnormalities are not sufficient for stabilizing MGUS.

(3) The sole tissue disruption could generate abnormal proliferation and stabilization of MGUS without the pre-existence or appearance of genetic alterations in the expansion phase. The lack of full differentiation due to the lack of possibility to reside in the niche is sufficient to produce residual proliferation and phenotypic instability. This would allow cells to explore new phenotypes that could ultimately lead to transformation without specific and identifiable “driver” genetic alteration. Genetic changes would appear later because of a global destabilization of the cells [63,65].

## 4. Perspectives for the Management of MM

By and large, the new concept of TiDiS gives BME a role in the initiation and promotion of cancer in general and in MM in particular, through disruption between cells and the BME. In this context, BME appears as a therapeutic target as well as cells being targets in the standard approach. More precisely, according to the TiDiS concept, a goal of the treatment will be to act on the BME to “re-educate” it, in order to restore the cellular interactions present in the initial tissue, so as to control cells by restabilizing their phenotypes. Much experimental evidence indicates that the normal microenvironment is able to control genetically altered cells and reduce phenotypic plasticity [21,63,64,65,71]. Thus, mimicking this normal micro-environment with molecules that would mimic healthy cellular interactions, together with molecules stimulating the re-expression of the proteins necessary to interact with these partners, would be efficient in stopping cell proliferation and cancer evolution [60,74].

In MM, this role is devoted to the BME since bone is the tissue of reference of this disease. According to the TiDiS concept, MM bone disease is not simply viewed as end damage, and bone tissue is not simply viewed as a permissive environment attracting MM cells and favoring MM cell growth through a now traditional and well-documented vicious circle. Disrupted bone tissue remodeling is implicated in the initiation and promotion of MM. In this context, this view implies the necessity of a better evaluation of this BME, not only at the stage of overt MM, but also earlier, at the stage of MGUS. In particular, a better evaluation of bone fragility in MGUS and of the degree of generalized bone loss in overt MM through quantitative histology and tomodensitometry appears necessary before attempting to correct them. The concept of “re-educating the BME” and the validity of this micro-environmental approach is already supported in MM by some clinical evidence [75]. Bisphosphonates, which are potent inhibitors of bone resorption, are efficient compounds in the management of MM, not only to reduce the occurrence of skeletal events but also to improve at least remission duration [76]. Furthermore, new anti-tumor drugs like proteasome inhibitors and immunomodulatory imide drugs are also effective because they act on the BME, especially proteasome inhibitors which are able to restore bone formation [77,78].

In the future, epigenetic-based treatments are clearly adequate for the first step that is to re-establish the initial interactions of MM cells with the BME. In MM, knowing the role of decorins and other interaction proteins in the inhibitory effects of osteoblasts over MM cells [79], providing decorins or “pseudo-decorins” as soluble proteins, and/or promoting osteoblast activity [80,81,82] would be good candidates for the second step [60], which is to mimic their inhibitory effects. Providing such peptides that mimic interactions domains of key environmental proteins could substitute for normal osteoblasts. Overall, the TiDiS concept outlines the BME and its interactions with MM cells (and precursor cells) as major therapeutic target. The restoration of a normal BME, bone formation in particular, appears as a major purpose in MM and its early stages. Until now, efforts have been mainly made on the inhibition of bone resorption using bisphosphonates or new compounds (reviewed in [83]), which appears insufficient. In this context, the treatment of MM and its precursor stages could take advantage of progress accomplished in the treatment of osteoporosis.

## 5. Conclusions

Although bone disease is a hallmark of MM, it is not included into the standard model of MM oncogenesis. Indeed, this standard model is mainly based on the genetic alterations occurring in the precursors of MM cells and turns out to be a “chromosome- and gene-centric” one. In this model, bone disease is simply viewed as an end damage of MM. However, more recent views insist on the role of a vicious circle between bones and MM cells to favor MM cell growth, and on the permissive role of the BME to attract the precursors of MM cells and to favor their development inside the bone marrow. Thus, the role of the MM BME could be more important than expected, especially during the early stages of the disease. The TiDiS hypothesis offers a new vision of the role of the BME in the occurrence of MM and of its precursor stages, beyond the 3 traditional views of MM bone disease: “End damage”, “vicious circle”, and “permissive”. It considers BME, and especially bones, as the tissue of reference in MM and suggests that its disruption plays a role in the initiation and promotion of MM, by favoring phenotypic and genetic instability within MM cells and their precursors.

This view impacts the management of MM and of its precursor stages. Until now, therapeutic efforts have been mainly made to reduce MM cell mass in overt MM and to limit the excessive bone resorption induced by MM cells thanks to the use of bisphosphonates as soon as the early stages of MM occur. According to the TiDiS concept, the BME, especially the disrupted endosteal niche, now appears as a major therapeutic target, as soon as the MGUS stage occurs. The evaluation of such an early bone disruption, mainly characterized by an early deficiency of bone formation, and its treatment in a similar way to that of osteoporosis, could prevent the occurrence of MM from MGUS or prevent the generalized bone loss observed in overt MM.

In the future, more works will be necessary for a better evaluation of the TiDiS concept, especially through the use of animal models (5T2), in order to propose a complete model of MM oncogenesis.

## Figures and Tables

**Table 1:** Main correlations between genetics, epigenetics, differentiation status, natural history, and micro-environment.

Chromosomal Abnormalities and Ploidy	Chromosomal Instability	Differentiation Status	Natural History: MGUS Phase Duration	Relation to Micro-Environment
t(11;14), diploid	Low (33% of incidence of -13q)	Full differentiation	Long	Close to normal
t(4;14), non-hyper-diploid	High (85%)	Lack of differentiation	Short	Strongly disrupted
Trisomy (1, 3, 5, 7…), hyper-diploid	Just right (50%)	Intermediate differentiation	Intermediate	Vicious dependence

**Table 2:** Different models of multiple myeloma (MM) oncogenesis according to their initiating and promoting events.

Model of MM Oncogenesis	Standard	Unifying	Extended and Permissive	TiDiS
Initiation level	Cell	Cell	Cell	Tissue
Initiating event	Genetics	Genetics	Genetics	Microenvironment
Promoting event		Epigenetics	Epigenetics + microenvironment	Genetics + epigenetics
Supporting features of MM cells and their precursors	Genotype	Morphotype, phenotype, proliferation index	Extended phenotype to bones (LBL, generalized bone loss)	Abnormal cellular interactions within bone

**Table 3:** Cellular and tissue events favoring a microenvironmental-based model of MM.

Cellular Events (Seeds): Natural History of MM	Pre-MGUS Stages: Reactive Plasmacytoses, Transient MGUS	Permanent MGUS (Transition from Transient MGUS)	Overt MM (Transition from Permanent MGUS)
Tissue events (soil): Mechanisms	Physiological disruption of the mesenchymal stromal to osteoblast transition	Disruption of the endosteal niche, shift from osteoblastic to osteoclastic status	Uncoupling between bone resorption and formation accentuation of the pre-existing disruption and shift of the bone remodeling complex
Consequences	Bone senescence	Generalized bone loss (bone fragility) through accentuation of bone senescence	Lytic bone lesions Generalized bone loss
